# Internal traction combined with pre-suturing assisted endoscopic submucosal dissection for the treatment of a giant polypoid lipoma of the terminal ileum

**DOI:** 10.1055/a-2901-6601

**Published:** 2026-07-13

**Authors:** Yang Yang, Fu Guan, Taofeng Jiang, Fuqun Wang

**Affiliations:** 1 Department of Gastroenterology 608523 Meizhou People’s Hospital, Meizhou Academy of Medical SciencesMeizhouGuangdong ProvinceChina

**Fig. 1:**
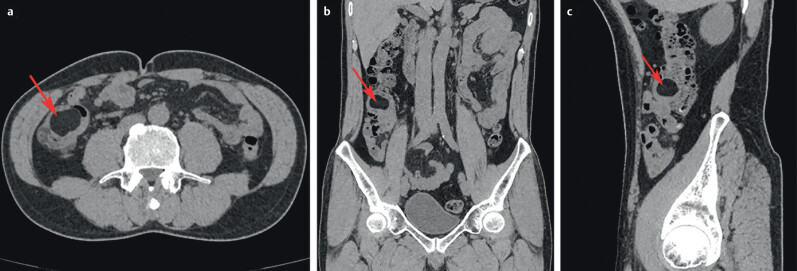
Computed tomography revealed a benign space-occupying lesion measuring approximately 4.0×3.5 cm in the terminal ileum: ( **a** ) an axial plane, ( **b** ) a coronal plane, and a ( **c** ) sagittal plane.

**Fig. 2:**
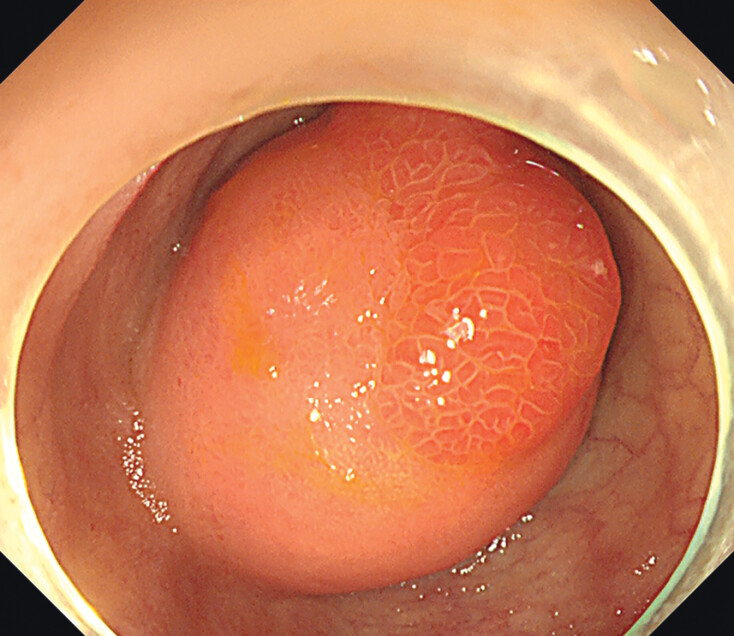
Colonoscopic examination revealed a large polypoid mass in the terminal ileum.

**Fig. 3:**
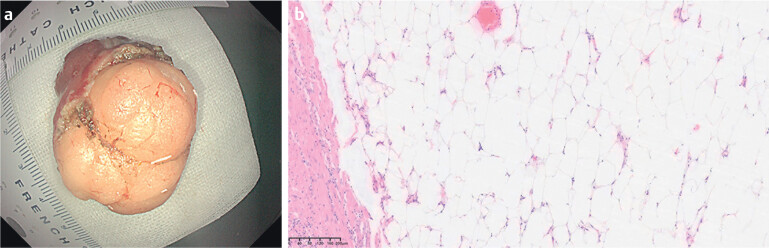
Postoperative specimen and histopathological examination. ( **a** ) The resected lesion and ( **b** ) histopathological examination confirmed the diagnosis of the lipoma (hematoxylin–eosin staining, 100×).

A 58-year-old male patient was referred to our hospital following the detection of a terminal ileal polyp via colonoscopy at the initial hospital, where he presented with recurrent abdominal pain lasting 1 month. Upon admission, computed tomography revealed a benign space-occupying lesion measuring approximately 4.0 × 3.5 cm in the terminal ileum ( Fig. 1 ). Colonoscopy demonstrated a large polypoid mass located 40 cm proximal to the ileocecal valve in the terminal ileum, characterized by a soft texture and hyperemic surface ( Fig. 2 ). The terminal ileum is characterized by a thin intestinal wall, abundant blood supply, frequent peristalsis, limited space, and difficulty in exposing lesions, which renders endoscopic submucosal dissection (ESD) in this region extremely challenging. 1​ 2​ ​3​ The patient's lesion was successfully and completely resected via the ESD assisted by internal traction and pre-suturing ( Video 1 ). During the procedure, the mass was initially secured to the ascending colon via internal traction using a self-made rubber band combined with titanium clips. Submucosal injection was administered along the lesion base, followed by circumferential mucosal incision to expose the submucosal layer. Upon encountering suboptimal cutting line conditions during submucosal dissection, the internal traction on the anal side was released, and the lesion was repositioned and fixed to the oral side using the same rubber band-titanium clip internal traction method. Submucosal dissection was subsequently continued along the lesion capsule. Prior to the complete transection of the lesion, interrupted pre-suturing with titanium clips was performed incrementally from the oral side to the anal side of the wound bed. The lesion was then transected after partial closure of the wound. The postoperative specimen was histopathologically confirmed to be a lipoma ( Fig. 3 ). The patient was discharged uneventfully on the second postoperative day. No recurrence was observed during a follow-up period of over 1 year.

**Video 1** Internal traction combined with pre-suturing assisted endoscopic submucosal dissection for the treatment of a giant polypoid lipoma of the terminal ileum.


Endoscopy_UCTN_Code_TTT_1AQ_2AD
